# Estimating Uninsured and Underinsured Women Eligible for Minnesota’s Breast Cancer Screening Program

**DOI:** 10.21203/rs.3.rs-2886477/v1

**Published:** 2023-05-16

**Authors:** David Haynes, Kelly D. Hughes, Maria Borerro, McKenna Haas, Lauren Roach, Anne Blaes

**Affiliations:** University of Minnesota; Minnesota Department of Health; University of Minnesota; University of Minnesota; University of Minnesota; University of Minnesota

**Keywords:** small area estimates, mammography services, health geography, prevention

## Abstract

The mission of the National Breast and Cervical Cancer Early Detection Program’s (NBCCEDP) mission is to improve access to mammography and other health services for underserved women. Since its inception in 1991, this national program has improved breast cancer screening rates for women who are uninsured and underinsured. However, the literature has shown that NBCCEDP screenings are decreasing, and only reach a portion of eligible women. Reliable estimates at the sub-county level are needed to identify and reach eligible women. Our work builds upon previous estimates by integrating uninsured and insurance status into spatially adaptive filters. We use spatially adaptive filters to create small area estimates of standardized incidence ratios describing the utilization rate of NBCCEDP services in Minnesota. We integrate the American Community Survey (2010–2014) insurance status data to account for the percentage that an individual is uninsured. We test five models that integrate insurance status by age, sex, and race/ethnicity. Our composite model, which adjusts for age, sex, and race/ethnicity insurance statuses, reduces 95% of the estimation error. We estimate that there approximately 49,913.7 women eligible to receive services for Minnesota. We also create small geography (i.e., county and sub-county) estimates for Minnesota. The integration of the insurance data improved our utilization estimate. The development of these methods will allow state programs to more efficiently use their resources and understand their reach.

## 1. Introduction

The National Breast and Cervical Cancer Early Detection Program (NBCCEDP) is a national program administered by the Centers for Disease Control that provides funding for breast cancer screening for low-income women (Health et al., 1995). Early detection of breast cancer through screening is critical to finding and treating cancer early when treatment is more likely to be successful (Ginsburg et al., 2020). The program has provided over 15.7 million mammograms and annually provides screening services for over 250,000 women (Centers for Disease Control and Prevention, 2022). However, a gap in the literature has been determining at the national and local levels how many women are eligible to receive NBCCEDP services.

Tangka et al. first described the nation’s state-level estimates for NBCCEDP breast cancer screening services (Tangka et al., 2006). They estimated the numbers of women aged 40–64 at both the state and national levels and the percentage of those who received those services using census population data. Howard and colleagues (2015) updated these national estimates, by integrating the Current Population Survey to specifically estimate women’s uninsured status by race/ethnicity and age group. Their results compared NBCCEDP screening from 2002–2003 and 2011–2012. Nationally the NBCCEDP screened more women, but the number of eligible women increased dramatically lowering the proportion of women reached in the program. Subramanian and colleagues (2015) continued this work, by examining the effectiveness of the NBCDEEP state-run programs. A contribution of their work is the integration of Behavioral Risk Factor Surveillance System (BRFSS) data. The BRFSS mammography prevalence measure is used as a proximal measure for women who were current with mammography. In contrast to the national approaches, Hughes et. al (2021) used a geographic small-area estimation technique to estimate the number of women eligible for Minnesota’s NBCCEDP. This work provided estimates and utilization rates of mammography services for the sub-county level, a limitation of this work was its ability to provide reasonable uninsured estimates.

The lack of an accurate population description for uninsured and underinsured women has been a consistent problem for estimating the population eligible for NBCCEDP services. Creating reliable estimates is elusive because publically available insurance estimates are aggregated by geographic location, race, and income. The Small Area Health Insurance Estimates (SAHIE) provides reliable 1-year estimates at the county level but has not yet been used for creating NBCCEDP estimates. We address this gap in the literature through the integration of detailed uninsured and underinsured rates by age, race, and income. However, instead of integrating the one-year SAHIE data, we integrate the 5-year uninsured data from the American Community Survey (ACS), which are available for small areas (i.e., census tracts)

## 2. Methods

### 2.1. Study Context

The Minnesota NBCCEDP, “Sage” screens approximately 15,700 women a year, across a network of over 400 participating clinics covering most of the state of Minnesota. Women 40 years and older are eligible for breast cancer screening every year if they have income at or below 250% of the federal poverty level and are uninsured or underinsured. This analysis updates previously published results (Hughes et al., 2021). This project is a secondary analysis of observational data for the purpose of program evaluation and was determined exempt by Institutional Review Board.

### 2.2. Data

#### 2.2.1. Sage Mammography Data

Utilization of breast screening services through Sage was defined as the count of women screened every year divided by the potentially eligible population. The residential locations of Sage women screened between 7/1/2010 and 6/30/2015 (n = 74,712 representing 5 fiscal years of data) were retroactively geocoded by the Minnesota Department of Health. Previously published work by Hughes and colleagues describes the geocoding methods used for residential addresses with low geocode scores and how to place individuals with only residential address ZIP code information (Hughes et al., 2021). Non-Minnesota residents screened through Sage were not included in this analysis.

#### 2.2.2. Population Data

The Research Triangle Institute (RTI) 2010 U.S. Synthesized Population dataset was used as the base population from which we defined different eligible populations (Rineer & Bobashev, 2020). The synthetic population provides “individuals” with age, sex, and race information, as well as household size and income. Each individual is linked to a household and is provided a geographic location. Using this information, we can calculate the population estimates of women in Minnesota, 40 and older that meet Sage’s criteria for mammography screening.

#### 2.2.3. Health Insurance Data

We used the publicly available American Community Survey (ACS) 5-year estimates for 2010 to 2014 for estimates of individuals who have insurance. Insurance estimates are provided in three separate datasets by age-sex, race, and income (Manson, Steven et al., 2022). While breast cancer does affect both men and women for our analysis, we only used women for the age-sex insurance information and refer to it as age. ACS insurance estimates are available at both the census tract and zip code levels. We chose to use the ZIP Code Tabulated Areas (ZCTA) as uninsured estimates for this study. ZCTA was chosen as it would not bias the results as there were cases that only provided their zip code.

### 2.3. Integrating Health Insurance into Spatially Adaptive Filters

Spatially adaptive filters are a geographic small area interpolation technique that controls for errors in estimate calculation by using a population threshold (Beyer & Rushton, 2009; Haynes et al., 2022; Tiwari & Rushton, 2005). The filters grow in size to ensure that each area calculated has the minimum population. The reference grid and population threshold used for creating the spatially adaptive filters are the same as in our previous work (Hughes et al., 2021). We integrated health insurance into the adaptive filters by applying the percentage of each individual’s uninsured status by age, race, and income that is aligned to the ZIP code in which they reside.

To do this, we used the synthetic population dataset that facilitates integration with spatial and aspatial datasets. In our case, we needed to apply a probability to each eligible woman that they are likely to be uninsured. We can do this by spatially joining the woman’s residence to the ZCTA. Once they are linked, we assigned them the value of that ZCTA. The synthetic dataset reports each individual’s age, race, and income characteristics, which allowed us to assign the appropriate ACS insurance estimates for each of these categories. Each insurance category (i.e., age, race, income) is subdivided into additional sub-categories. The age category has five sub-categories (35 < 44, 45 < 54, 55 < 64, 65 < 74, < 75+), there are also five sub-categories for income (< 25K, 25K < 49K, 50K < 74K, 75K < 100K, < 100K) and six sub-categories for race (i.e., White-nonHispanic, Black, Asia, Pacific Islander, some other race, and two or more races). The synthetic population dataset supports the same race definitions but does not have a category for ethnicity. Therefore we align the synthetic population designated as White with the White-nonHispanic ACS insurance estimates. Aligning these categories allows us to provide precise insurance estimates for individuals based on their age, race, and income characteristics, and based on the ZCTA where each person resides.

**Figure 1:**
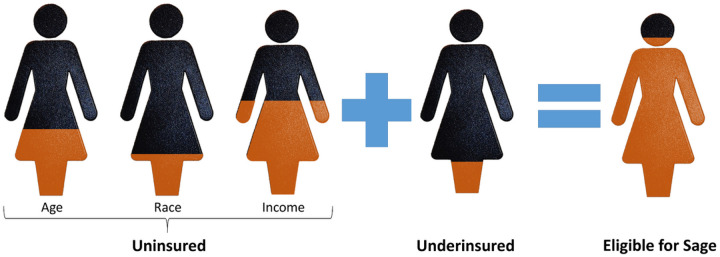
Illustrates the Calculating Uninsured and Underinsured for Sage

To determine the insurance estimates for the composite model, we simply add together for each individual the amount of uninsured by age, race, and income plus a constant of 0.1 for all individuals who do not identify as American Indian or Alaska Native (Fig. 1). Adding together each of the insurance categories allows for a comprehensive understanding of all insurance estimates. The constant we apply is a conservative estimate of women that are underinsured for SAHIE data (Schoen et al., 2011; United States Census Bureau, 2017). Grid points are interpolated into a surface using an inverse distance weight algorithm in ArcGIS Pro, using our previous approach (Haynes et al., 2022; Hughes et al., 2021).

## 3. Results

The model that we have developed allows us to simulate different insurance scenarios that will predict the population eligible for the NBCCEDP. Table 1 provides the summary number of women eligible to receive services. If you apply a constant for insurance, which we previously calculated at 22.6%, we estimate that there are approximately 36,874 women in Minnesota that the Sage program could screen (Hughes et al., 2021). Table 1 shows that integrating different insurance estimate models provides very different results. Integration of just the age-based insurance estimate has the smallest available (25,833) while the largest number available is the fully integrated or composite model with (49,913) women. The model results are smaller when it uses a single dataset (e.g., age, income, or race) as the composite model is additive.

**Figure 2:**
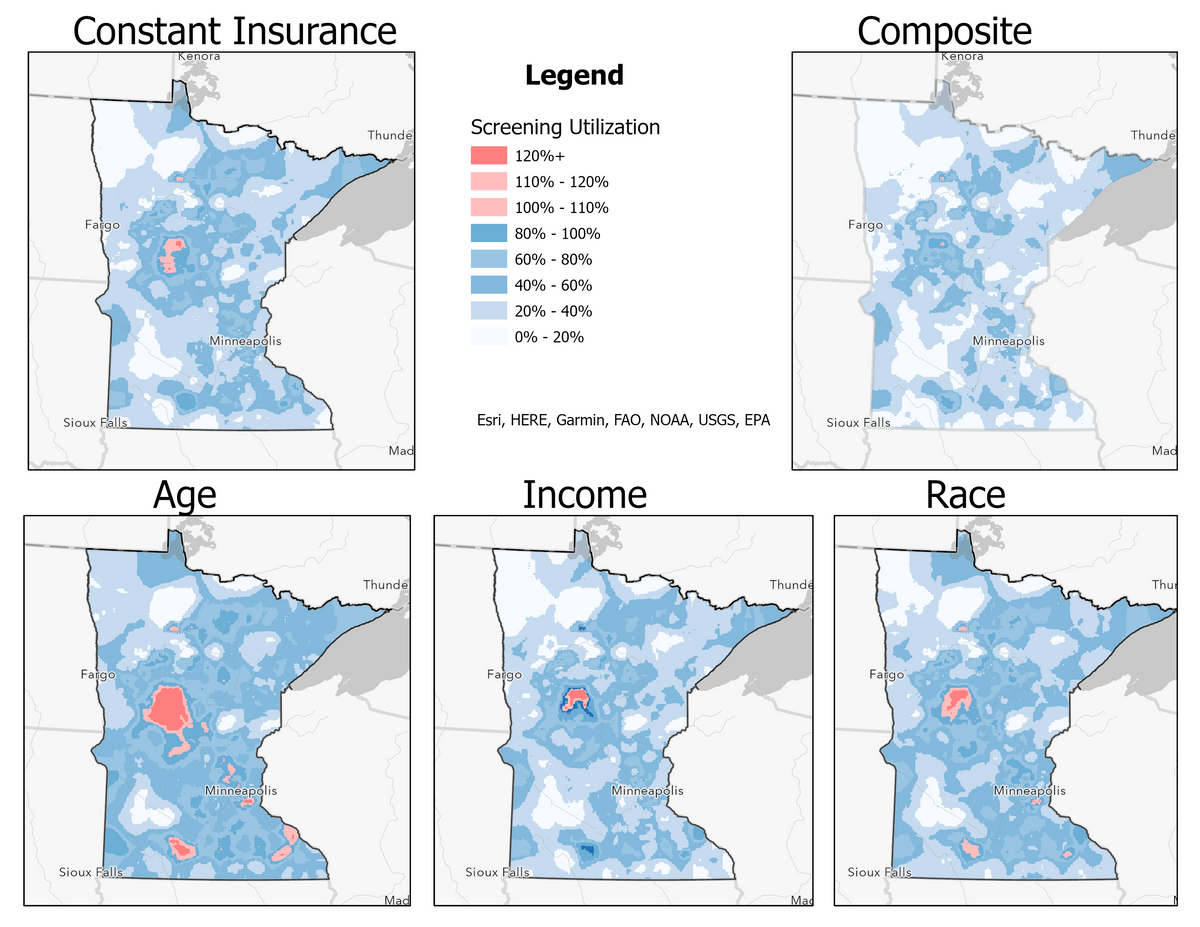
Utilization of Mammography Services with Differing Insurance Models

Figure 2 illustrates how these estimates appear when mapped. Additionally, we highlight in red any area where we have an underestimate. Figure 2 illustrates that all models have underestimated the number of women eligible for NBCCEDP services. The error varies in size, location, and magnitude. Figure 2A (Constant Insurance) shows that there is a localized underestimate in the west-central portion of the state. The error is primarily in the first two categories (100%−110% and 110%−120%). In Fig. 2B (Age), the error is no longer localized. In addition to the west-central error, new areas in the southeast portion of the state have been underestimated. Also, Fig. 2B has many more pixels that are in the largest error category of 120% and above. In comparison, Figs. 2C (Income) and 2D (Race) are substantial improvements to Fig. 2B (Age). The income model seems to have the most localized error in comparison to the other two insurance models. The race model (Figured 2D) has additional areas of underestimation in the south and southeast portions of the state.

When compared to all of the other models, Fig. 2E (Composite), which combines all of the insurance estimates, has the least error, upon visual inspection. Figure 2E also has the most similar utilization spatial patterns of all the new models, when compared to the original model (Figured 2A). In particular, there are low utilization areas in the northwest and southwest portions of the state in Fig. 2A. The composite model (Fig. 2E) reflects a similar spatial pattern for these same areas.

Table 2 provides summary statistics of the utilization rate for each of the models in Fig. 2. Overall, the models show that the state utilization rate of breast screening services varies from as low as 26.52% percent to as high as 53.93%. The largest standard deviation is attributed to the age model because this model had the lowest estimates of women eligible for the NBCCEDP program, which resulted in the largest underestimates.

We also identified how often our utilization was over 100%. All of the maps have some residual error. The constant insurance model had about 5% of its cells with a utilization rate of over 100%. The age, income, and race models all increased the total amount of error in the data set. This is due to the models underpredicting in particular areas the number of women eligible for the NBCCEDP. Of the three models, the income error has the least number of cells with a value over 100%.

These results also show that our composite model, which integrates all three insurance statuses for an individual, is the best model. It improves our estimates by reducing estimation error by 95.2%. The composite model has less than 1% of cells with any error. In comparison, the other three insurance models (i.e., age, income, race) increase the overall error. We estimate that 49,914 uninsured and underinsured women are eligible to receive services within the state of Minnesota.

**Figure 3:**
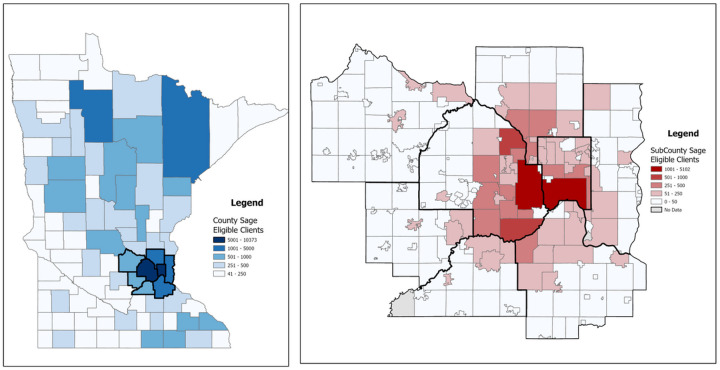
Aggregation of Estimated Women Eligible for NBCCEDP Services in Minnesota at Different Geographic Variations

While utilization rates are helpful for understanding the uptake of a particular resource, estimated counts are important for programmatic goal setting. The resulting eligible population uses synthetic geographic locations, and it can be spatially aggregated into geographic boundaries. Sage works with providers in every county in Minnesota, and Fig. 3A describes for each county the number of women underinsured or uninsured who are eligible to use Sage screening services. A detailed description of the entire state’s eligible population by county is included (Appendix 1). Figure 3A also illustrates the spatial variation of women eligible to receive services at the county level is distributed across urban and rural counties. The two counties (i.e., Hennepin and Ramsey) with the largest number of women eligible for NBCCEDP services are the two most populated counties in Minnesota. These two counties belong to the highest category meaning that over 5,000 women are eligible for NBCCEDP services in each county. The counties that are immediately adjacent (i.e., Wright, Anoka, Washington, Carver, Scott, and Dakota) are members of the second and third categories, meaning 501 to 5,000 women are eligible in each county. However, there are rural areas in the central northern portion of the state that also have a large number of women eligible for NBCCEDP services. The county of Beltrami, which has a large American Indian population has over 1,000 women eligible for NBCCEDP services. There are several counties (i.e., Becker, Otter Tail, Cass, Itasca, and Crow Wing) all located in the northern central portion of the state with at least 500 women eligible for NBCCEDP services.

County-level estimates may be effective for state-wide initiatives but insuffi cient for local clinic efforts. Figure 3B highlights the variation of eligible women in the seven-county metro area previously described. Figure 3B uses census county divisions (CCDs), which are established geographic boundaries cooperatively developed by the Census Bureau and state and local governments (Federal Register, 2018). County division units were chosen over ZCTA as our models integrated insurance data at the ZCTA level. The CCDs further highlight the spatial variation of individuals eligible for NBCCEDP services at the sub-county level and the importance of spatial disaggregation. Hennepin and Ramsey counties each have a single CCD, which are the designated areas of Minneapolis and St. Paul, belonging to the highest category that has over 1,001 individuals eligible for NBCCEDP services. The visual-spatial pattern present in Fig. 3B indicates that there is a trend, in which CCDs that have large numbers of women eligible for NBCCEDP services tend to be near similar CCDs.

## 4. Discussion

Our work builds upon previous work by Hughes and colleagues (Hughes et al., 2021) by integrating insurance status into spatially adaptive filters. The integration of these datasets has reduced the error in our original model by 95.2% and resulted in less than 1% error remaining in our mammography utilization estimates. Additionally, this work establishes small-area estimates for a state NBCCEDP. These methods fill a critical gap in the literature by providing detailed high-spatial resolution estimates at multiple geographic levels designating the number of women eligible to receive NBCCEDP resources. This provides a foundation for future research, which can then further examine the personal and structural barriers that have been eliminated that allow for high utilization rates of mammography services in particular communities.

Mammography rates continue to be lower among low-income women and racial and ethnic minorities (Davis et al., 2014). Howard (2015) estimated that while the total number of women using the NBCCEDP program had increased, the overall percentage had decreased (Howard et al., 2015). This is why creating a reliable estimate of women eligible for the NBCCEDP program is critical. The NBCCEDP serves a critical role in providing access to mammography for low-income women and racial and ethnic minorities, who often have irregular access to healthcare (Centers for Disease Control and Prevention, 2019; NBCCEDP Screening Program Summaries, 2022). Women who do not have regular access to care, often delay mammography, which has a rippling effect on underserved populations and ultimately leads to worse survival and outcomes (Mootz et al., 2020). These underserved populations are more likely to be diagnosed with advanced cancer, in part due to missed opportunities for timely screening. In the US, African-Americans are more than twice as likely, and Hispanics are 1.2 times as likely to be diagnosed with metastatic disease than non-Hispanic whites (Clegg et al., 2009).

### 4.1. Limitations

Our work only created estimates for one state’s NBCCEDP and therefore it is unknown how generalizable the model is to other states. Future work should extend this research to multiple states to determine the generalizability of the model. Additionally, the estimates are based on the 2010 population and may need to be updated. However, the RTI synthetic population dataset that was used in this data is no longer freely available for the 2020 census.

### 4.2. Conclusions

Our geographic small area estimation approach demonstrates one potential critical step for accurately characterizing the population eligible for NBCCEDP services at the sub-county level. In particular, mammography utilization maps can be used by state offi cials to evaluate the effi cacy of partnered clinics and plan interventions in communities. The estimates and maps are relevant resources that public health decision-makers could use for the deployment of limited resources and evaluation of the program. This work estimates the women eligible and demonstrates the utilization rates of mammography resources at the sub-county level.

## Figures and Tables

**Table 1:** Total Minnesota NBCCEDP Eligible Population

Insurance Integration Approach	Women Eligible
Constant 22.6% Insurance	36,874.80
Age	25,833.79
Income	38,074.91
Race	31,024.65
Composite	49,913.76

**Table 2:** Summary Statistics of Interpolated Utilization Rates Using Different Insurance Models

	Minimum Utilization Rate	Maximum Utilization Rate	Mean Utilization Rate	Standard Deviation	Percent of Over Estimated Cells greater than 100%	Percent of Error Changed Compared to Constant Insurance Model
Constant	0.00	131.11%	37.18%	0.187	5.30%	---
Age	0.00	176.88%	53.93%	0.255	31.65%	+ 497.11%
Income	0.00	141.38%	37.19%	0.187	6.98%	+ 31.75%
Race	0.00	141.21%	43.83%	0.205	10.01%	+ 93.60%
